# Cost-effective priorities for the expansion of global terrestrial protected areas: Setting post-2020 global and national targets

**DOI:** 10.1126/sciadv.abc3436

**Published:** 2020-09-09

**Authors:** Rui Yang, Yue Cao, Shuyu Hou, Qinyi Peng, Xiaoshan Wang, Fangyi Wang, Tz-Hsuan Tseng, Le Yu, Steve Carver, Ian Convery, Zhicong Zhao, Xiaoli Shen, Sheng Li, Yaomin Zheng, Han Liu, Peng Gong, Keping Ma

**Affiliations:** 1 Institute for National Parks, Tsinghua University, Beijing, China.; 2 Department of Landscape Architecture, School of Architecture, Tsinghua University, Beijing, China.; 3 Ministry of Education Key Laboratory for Earth System Modeling, Department of Earth System Science, Tsinghua University, Beijing, China.; 4 Ministry of Education Ecological Field Station for East Asian Migratory Birds, Beijing 100084, China.; 5 Wildland Research Institute, School of Geography, University of Leeds, Leeds, UK.; 6 Centre for National Parks and Protected Areas, University of Cumbria, Cumbria, UK.; 7 Institute of Botany, Chinese Academy of Sciences, Beijing, China.; 8 School of Life Sciences, Peking University, Beijing, China.; 9 State Key Laboratory of Remote Sensing Science, Aerospace Information Research Institute, Chinese Academy of Sciences, Beijing, China.

## Abstract

Biodiversity loss is a social and ecological emergency, and calls have been made for the global expansion of protected areas (PAs) to tackle this crisis. It is unclear, however, where best to locate new PAs to protect biodiversity cost-effectively. To answer this question, we conducted a spatial meta-analysis by overlaying seven global biodiversity templates to identify conservation priority zones. These are then combined with low human impact areas to identify cost-effective zones (CEZs) for PA designation. CEZs cover around 38% of global terrestrial area, of which only 24% is currently covered by existing PAs. To protect more CEZs, we propose three scenarios with conservative, moderate, and ambitious targets, which aim to protect 19, 26, and 43% of global terrestrial area, respectively. These three targets are set for each Convention on Biological Diversity party with spatially explicit CEZs identified, providing valuable decision support for the post-2020 global biodiversity framework.

## Teaser

Cost-effective zones for the expansion of global terrestrial protected areas are identified.

## INTRODUCTION

Global biodiversity is declining faster than at any time in human history (*1*–*3*), with potentially dire consequences for human society (*4*). Protected areas (PAs) are the cornerstones of biodiversity and conservation (*5*). In 2010, parties to the Convention on Biological Diversity (CBD) proposed 20 Aichi targets to prevent biodiversity loss, with Target 11 specifically calling for PAs to be increased and improved [by 2020, at least 17% of terrestrial and inland water and 10% of coastal and marine areas are conserved through effectively and equitably managed, ecologically representative and well-connected systems of PAs and other effective area-based conservation measures (OECMs)]. Since then, coverage of terrestrial PA has grown from 12.7% in 2010 to 15.2% in 2020, which may continue to grow according to future commitments from CBD parties (*6*). However, the current global PA network has not successfully mitigated the ongoing decline of biodiversity and ecosystem services (*6*, *7*), and there is overwhelming agreement that Aichi Target 11 is not adequate to conserve biodiversity (*8*).

The 15th Meeting of the Conference of the Parties to the Convention on Biological Diversity was planned to be held in Kunming, China, in October 2020 (which is postponed because of the coronavirus disease 19 pandemic). The conference is themed around “Ecological Civilization: Building a Shared Future for All Life on Earth,” and the final decision on the post-2020 global biodiversity framework will be made at this meeting. According to the zero draft of the post-2020 global biodiversity framework (*9*), a global, outcome-oriented framework should be provided for the development of national goals and targets, in which protection of sites of particular importance for biodiversity through PAs and OECMs is still an emphasis. In addition, a “no loss” goal was proposed toward those critical ecosystems that are rare, vulnerable, or important (*10*). It is obvious that within the post-2020 framework, coverage targets for global and national PA are crucial and should cover those critical ecosystems to the best, which in turn gives rise to the urgent question: “Where are the most effective and feasible regions for PA designation to protect biodiversity cost-effectively?” Previous studies provide much of the required research basis to help answer this question. Several studies have identified the priority areas for biodiversity conservation, including Crisis Ecoregions (CEs) (*11*), Biodiversity Hotspots (BHs) (*12*), Endemic Bird Areas (EBAs) (*13*), Key Biodiversity Areas (KBAs) (*14*), Centers of Plant Diversity (CPDs) (*15*), Global 200 Ecoregions (G200s) (*16*), and Intact Forest Landscapes (IFLs) (*17*). These templates of global biodiversity conservation prioritization are widely recognized and represent several important facets of biodiversity conservation. However, the identified regions invariably also include areas with high human impact (e.g., cities and farmland), which makes designating PAs much more difficult.

As a result, the targets set by conservation scientists often do not align with political objectives or policy goals (*18*, *19*). However, there have also been several studies that have identified wilderness areas with lower levels of human impact, where PA designation in line with Aichi Target 11 is both suitable and feasible (*20*–*23*). These studies also indicate that minimizing human disturbance could enhance the biodiversity conservation effectiveness of newly designated PAs. Although wilderness areas may not always offer the most effective biodiversity conservation opportunities (*5*, *24*), the effects of location and scale are important (*25*, *26*). For example, wilderness areas provide a buffering effect against species loss; the extinction risk for species within wilderness communities is on average less than half that of species in nonwilderness communities (*27*). Furthermore, while cost-effectiveness has been addressed in several studies (*28*, *29*), few have conducted comprehensive analyses to identify potential PAs with clearly defined spatial boundaries for each CBD party.

To summarize, there is a pressing need to understand where best to locate future PAs to maximize effectiveness and feasibility for biodiversity conservation. There is also a broad acknowledgment that Aichi Target 11 is not adequate to conserve biodiversity and a global protection of around 30 to 70% (or even higher) of Earth is well supported in the literature (*30*). For example, a target of nearly 28% has been put forward to conserve the entire terrestrial species, ecoregions, Important Bird and Biodiversity Areas, and Alliance for Zero Extinction Sites (*31*). In addition, 31% has been set as the bottom line for the post-2020 target for the conservation of globally important areas for biodiversity and ecosystem services such as carbon storage (*32*). Beyond that, the Nature Needs Half initiative (*33*, *34*) and Half-Earth vision (*35*, *36*) call to protect as much as 50% of the world and to protect at least 85% of the species on Earth. While the above studies propose (arguably laudable) post-2020 PA coverage targets, they lack the sufficiently high-resolution spatial planning for effective PA expansion; thus, the most cost-effective potential sites may not be designated. In addition, previous studies mainly focused on global headline targets, with fewer studies giving consideration for national targets or taking differentiated regional natural and social conditions into account.

To fill this knowledge gap and provide decision support for the development of the post-2020 global biodiversity framework (*37*), this study focuses on the spatial planning of global terrestrial PAs by identifying cost-effective priorities and setting global and national coverage targets. Four criteria are included: (i) the effectiveness in biodiversity conservation; (ii) the feasibility for PA designation that is both spatially explicit and high resolution, which requires the identification of target regions with clearly defined spatial boundaries; (iii) the different scenarios and priorities for policy-makers; and (iv) the heterogeneity for and within different countries. By considering the above criteria, this spatial planning aims to bridge the gap between conservation science and the political rationale required for the post-2020 targets.

## RESULTS

### Conservation priority zones

**Fig. 1:**
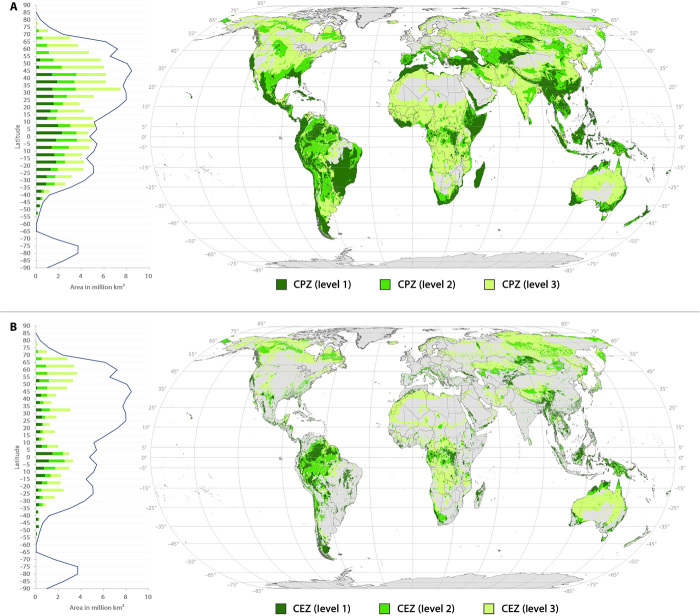
Global distribution of CPZs and CEZs. (**A**) distribution of CPZs. (**B**) distribution of CEZs. Charts on the left show the latitudinal distribution of CPZs and CEZs, in which polylines represent the total land area at the corresponding latitude.

Figure 1A maps the distribution of conservation priority zones (CPZs) by overlaying seven global biodiversity templates (figs. S1 and S2). Globally, CPZs cover 77.2% of the terrestrial area, including almost all terrestrial area near the equator (between 15°N and 15°S). However, most deserts and some areas of high northern latitudes are not identified as CPZs. These include the Australian Desert, Arabic Peninsula, Sahara, Taklimakan, and Russian Far East. Large areas of the European Plain, with a high level of human impact, are not identified as CPZs.

CPZs are classified into three levels according to the number of times they are identified by the seven global biodiversity templates. In terms of area, level 1, 2, and 3 CPZs take up 19.2, 19.1, and 38.9%, respectively, of global terrestrial lands. Level 1 CPZs, with the highest priority for biodiversity conservation, are mainly located in low and middle latitudes, including northern and eastern South America, East and Southeast Asia, eastern Africa, north of the Middle East, and southern North America. Level 2 CPZs usually surround level 1 CPZs, which are mainly located in South America, South Asia, and southern North America. Level 3 CPZs are widely distributed in Asia, North America, central Africa, and central Oceania.

### Cost-effective zones for PA designation

Figure 1B maps the distribution of cost-effective zones (CEZs) for PA designation, which are defined as CPZs under low levels of human impact. CEZs cover 37.8% of Earth’s land surface with level 1 covering 7.5%, level 2 covering 9.5%, and level 3 covering 20.8%. Low human impact areas (LIAs) cover 54.9% of terrestrial area (excluding permanent ice and snow), 68.9% of which are covered by CEZs, indicating that nearly two-thirds of LIAs have a high priority for conservation.

The coverage of CEZs is far less extensive than CPZs in middle and low latitudes, especially in eastern South America, South and Southeast Asia, eastern Africa, and Madagascar, while in high latitudes such as northern Asia and northern North America, the distribution of CEZs and CPZs is almost the same. This is due to the nonstationary distribution of human impact.

In terms of the distribution of different CEZ levels, level 1 CEZs are mainly located near the equator, including northern South America, Southeast Asia, and central Africa. Level 2 CEZs are mainly distributed in northern South America, Southeast Asia, northern Asia, northern North America, and central Africa. Level 3 CEZs cluster in high latitudes of the Northern Hemisphere, central Africa, and central Oceania.

### Global PA coverage targets

**Fig. 2:**
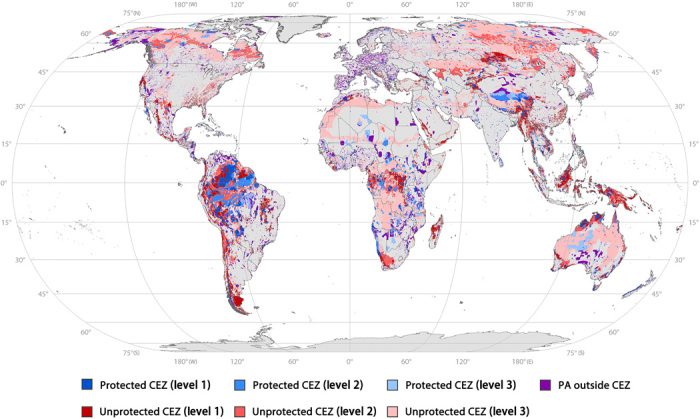
Global distribution of CEZs and existing PAs. CEZs uncovered by existing PAs (red) are considered highly feasible for PA expansion. The darker the color, the higher the priority.

Figure 2 maps the distribution of CEZs and existing PAs, showing the specific locations of unprotected CEZs with spatially explicit and clear boundaries. Large areas of CEZs are unprotected globally. For example, in northern South America, which is an important area for global biodiversity, there are still many unprotected level 1 and level 2 CEZs despite relatively good existing PA coverage. In northern Asia, the existing PA coverage is quite limited, leaving many level 2 and level 3 CEZs unprotected, while in Europe, the existing PAs are usually located outside CEZs.

Although 14.1% of the terrestrial area has already been designated as PAs globally (*38*), only 24% of CEZs are under protection, leaving the remaining 76% of CEZs unprotected. Filling these conservation gaps will not only increase the PA coverage in number but also promote the effectiveness of conservation in the suitable places, which will enhance the quality of the PA system.

The global targets under conservative, moderate, and ambitious scenarios require 19, 26, and 43% of total terrestrial area to be protected, respectively. The ambitious target is between 30 and 50% (*39*), echoing the Nature Needs Half initiative (*33*) and the Half-Earth vision (*35*). The moderate target is between 20 and 30%, and the conservative target is slightly higher than the 17% Aichi Target 11.

To achieve these targets, more CEZs should be protected where human impact is low and, thus, the cost of designating PAs is relatively low, while the target areas corresponding to the three scenarios have different conservation priorities. To achieve the conservative target, all unprotected level 1 CEZs should be conserved, which are areas of the highest conservation priorities for global biodiversity, and thus, strict conservation measures should be taken. To achieve the moderate target, in addition to unprotected level 1 CEZs, unprotected level 2 CEZs should also be protected to cover areas with medium conservation priorities. To achieve the ambitious target, all unprotected level 1, 2, and 3 CEZs should be protected, and more inclusive conservation measures could be considered. For practical purposes, we call for immediate actions to achieve the conservative target by conserving unprotected level 1 CEZs and using the moderate target as a medium-term goal for PA expansion by 2030 and the ambitious target as a longer-term goal by 2050. PA coverage targets for each continent are shown in fig. S3.

### National PA coverage targets

**Fig. 3:**
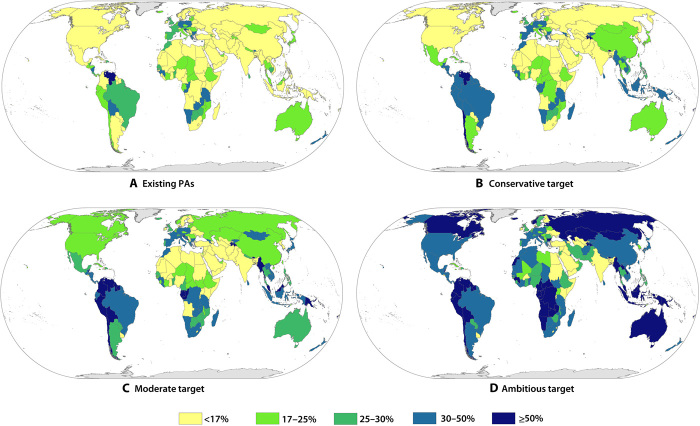
Maps of countries with different percent range protected under four scenarios. (**A**) existing PAs, (**B**) conservative target, (**C**) moderate target, and (**D**) ambitious target. All countries and regions (excluding Antarctica and Greenland) are considered. Note that, although the WDPA data are the best available, they may not include all PAs, which will cause underestimates of the existing PAs in certain countries.

We classified 195 of 196 CBD parties (not including the European Union) into five categories according to the percent range protected under different scenarios (Fig. 3 and Table 1). Detailed results for each CBD party are listed in table S1, including PA coverage targets in different scenarios (existing PAs and ambitious, moderate, and conservative targets), CPZ coverage, unprotected CPZs, CEZ coverage, and unprotected CEZs.

**Table 1:** Numbers of countries with different percent range protected under four scenarios. The total number and proportion of 195 CBD parties (not including the European Union) are divided into five categories according to percent range protected.

**Percent range protected**	**Scenarios**			
	**Existing PAs**	**Conservative target**	**Moderate target**	**Ambitious target**
**[0%,17%)**	109 (55.9%)	76 (39.0%)	64 (32.8%)	42 (21.5%)
**[17%,25%)**	42 (21.5%)	43 (22.1%)	32(16.4%)	17 (8.7%)
**[25%,30%)**	17 (8.7%)	13 (6.7%)	23 (11.8%)	31 (15.9%)
**[30%,50%)**	24 (12.3%)	49 (25.1%)	48 (24.6%)	57 (29.2%)
**[50%,100%]**	3 (1.5%)	14 (7.2%)	28 (14.4%)	48 (24.6%)

We recognize that individual countries are likely to play different roles in the projected global expansion of PAs. The top 10 countries with the largest PAs and highest PA coverage under the ambitious target are shown in fig. S4. Overall, the top 10 countries (including the Russian Federation, Australia, Canada, Brazil, China, the United States of America, Congo, Kazakhstan, Indonesia, and Angola) with the largest PA expansion potential contribute 66% to the global expansion of PAs under the ambitious target (fig. S5).

## DISCUSSION

### Policy implications at international and national levels

We have identified CEZs for future PA designation and proposed PA coverage targets at three scenarios at both global and national levels (table S1). As there is huge potential to add additional CEZs to the existing global PA network, CBD parties have the responsibility to protect more CEZs for effective biodiversity conservation and sustainable development.

At the international level, our research could be useful in developing the post-2020 global biodiversity framework. CEZs are sites of particular importance for biodiversity and feasible areas for designation of PAs; thus, protecting CEZs could help achieve the goals and targets proposed in the post-2020 framework. It should be also noted that, in achieving bold conservation targets and to maximize the conservation of CEZs, OECMs should also be considered as supplementary to PAs, which can provide positive conservation outcomes and have an important role in supporting coexistence, compatibility, and connectivity as part of an integrated approach to in situ conservation (*40*, *41*).

At the national level, our research may help policy development when considered as a part of a systematic conservation planning approach (or similar), e.g., in devising aligned legal and regulatory mechanisms spanning across various scales and jurisdictions to enable countries to update their National Biodiversity Strategies and Action Plans in a holistic, evidence-based manner. Previous targets for PA coverage have typically been discussed at the global level rather than being grounded in the realities of national/regional contexts (*42*, *43*). There are clearly important natural and social issues that need to be accounted for at the national level, where conservation needs are likely to be correspondingly different (*44*). The responsibility toward global biodiversity conservation (*45*), the demand and suitable areas for PA expansion (*31*), and the level of biodiversity under threat (*46*) can vary markedly between nations. If PA targets continue to operate solely at the global level, then there is a risk that even if the global targets for increasing PA coverage are achieved, this expansion may not align with the most effective potential areas, thus leaving many important areas unprotected. In this study, we highlighted the notable variations among countries in the potential contribution to global biodiversity conservation, indicating a need to consider country-specific targets with an overarching global target. Besides the numerical targets, we identified CEZs with relatively clearly defined spatial boundaries and different levels of conservation priorities, which are useful in stage planning with different conservation measures.

### Countries require special attention

On the basis of our research, there are five categories of countries that require special attention. These are as follows:

1) Mega CEZ/CPZ countries and megadiverse countries. These countries are crucial to global biodiversity conservation. CEZs are concentrated in a small number of countries including the Russian Federation, Australia, Canada, Brazil, China, and the United States of America, which together make up 53% of all CEZs by area and have the greatest potential for PA expansion. In addition, CPZs in eight countries (the Russian Federation, China, Brazil, the United States of America, Australia, Canada, India, and Argentina) account for 50% of all CPZs by area (fig. S6). Megadiverse countries are among the world’s richest for living organisms (*47*). The CPZs and CEZs of 17 megadiverse countries (including Australia, Brazil, China, Colombia, the Democratic Republic of the Congo, Ecuador, India, Indonesia, Madagascar, Malaysia, Mexico, Papua New Guinea, Peru, Philippines, South Africa, the United States of America, and Venezuela) account for 42.8 and 40.2% of global CPZs and CEZs by area, respectively, indicating the importance of these countries in global biodiversity conservation. However, the conservation status of CEZs varies greatly among these countries, with protected CEZ percentages ranging from 2.8% for Papua New Guinea to 66.0% for Venezuela. The potential for the expansion of PAs and associated targets therefore differs markedly among megadiverse countries (fig. S7).
2) Countries needing to protect more CEZs. These are countries with the largest unprotected CEZ areas globally or those with the largest area of unprotected CEZ as a percentage of their total terrestrial land area. The countries with the largest unprotected CEZs are largely consistent with the top 20 CEZ countries, except for Bolivia, which has already protected 42.2% of its CEZ areas (fig. S5). Countries with high proportions of unprotected CEZ areas should take immediate action to expand their PAs.
3) Countries with many CPZs but few CEZs. These countries not only have important biodiversity conservation value but also have substantial human activity. For example, CPZs account for 94.4% of the territorial area of India, but only 7.2% remain as CEZs. This indicates the potential for conflict between biodiversity conservation and human activity. Countries in this group are likely to require more inclusive conservation actions, such as using OECMs, and ecological restoration and/or rewilding.
4) Countries with many PAs but few LIAs or CEZs. As an example, Germany has 36.6% PA coverage of the land area, while CEZs only account for 3.1%. This indicates that countries with fewer LIAs can protect both biodiversity and cultural landscapes (e.g., traditionally farmed areas and their associated biodiversity) by establishing more inclusive PAs, and while not identified as CEZs at a global scale, these areas may have national and regional conservation significance. This also highlights that the targets we propose should not be seen as the upper limit of PA coverage; the PA system could be expanded outside CEZs to protect other areas with conservation values.
5) Non-CBD parties. The United States of America, as perhaps the most prominent nonsignatory to the CBD, is a megadiverse country, with 75.7% of its land area identified as CPZs. Its unprotected CEZs cover 18.9% of its land area and 4.6% of the world’s unprotected CEZs, indicating the potential for the expansion of the U.S. PA network and further contribution to global biodiversity conservation.

To summarize, seven countries are of top priority in terms of potential PA expansion, namely, Australia, China, Brazil, the United States of America, Kazakhstan, Indonesia, and the Democratic Republic of the Congo. It should also be noted that 19 countries have unprotected CEZs covering over 50% of their terrestrial area, most of which are less developed countries.

The effective implementation of the CBD requires clarification of each party’s rights and obligations. Countries undertake different responsibilities and face different challenges to achieve their national targets. The future socioeconomic development of countries with high PA coverage may be restricted, as large areas are set for conservation. The responsibility for biodiversity conservation in such countries should not be assumed independently but the common responsibility of the international community. This indicates that a global cooperation mechanism for the expansion of PAs is urgently needed; protecting biodiversity is both a shared responsibility of humankind and an economic imperative. Such multilateral global action could significantly improve the effectiveness of biodiversity conservation on a global scale (*3*, *45*), and as there are large national variations in the capacity to manage PAs effectively (*46*) and poorer countries tend to have lower capacity, often alongside high levels of biodiversity (*31*), we propose a global cooperation mechanism to share knowledge, good practice, and resources.

### Caveats and limitations

There are inevitably some uncertainties associated with this study, particularly those concerning data quality, which do need careful consideration. Despite using the best available data on global biodiversity templates, it was not possible to reflect the conservation need for all taxa and cover all aspects of biodiversity conservation, which may have led to an underestimation of CPZs. It was also impossible to exclude all human impacts, which may have led to an overestimation of LIAs. Although the World Database on Protected Areas (WDPA) represents the best available dataset, this database may not include all PAs, and data quality is often uneven across countries, which will cause underestimates of the existing PAs in certain nations (*48*). Because of these combined uncertainties, the PA coverage targets proposed in this paper may be either overestimates or underestimates, depending on the data quality in each country.

We recognize these limitations, and while our analysis is acceptable at an overarching global scale, the results need further validation and optimization using relevant data with higher resolution and accuracy in the future (*49*, *50*). In addition, the targets proposed for each CBD party in this study are only referential rather than mandatory, which provides a sound basis for parties to set their own formal targets and conduct the spatial planning of PAs by incorporating more national-scale datasets with higher accuracy and at finer resolution. It should also be noted that “how many protected areas are enough to conserve biodiversity” is still a challenging question and, thus, further studies are required on the basis of our results, which could be used as baseline data in the long-term planning and monitoring of global PAs.

## MATERIALS AND METHODS

### Identification of CPZs

We conducted a spatial meta-analysis of seven global biodiversity prioritization templates to identify the CPZs (*51*), including CEs, BHs, EBAs, KBAs, CPDs, G200s, and IFLs. The templates were then overlaid and categorized into three levels based on the number of times the zone is identified by different templates. Areas covered by three or more templates were defined as level 1 CPZs, those covered by two templates were defined as level 2 CPZs, and areas covered only by one template were defined as level 3 CPZs.

These templates were selected because (i) they identify important terrestrial regions in consideration of at least one facet of biodiversity; (ii) they are robust and widely used in global biodiversity modeling; and (iii) the data are relatively reliable and accessible. Explanations for each template are as follows: (i) CEs are ecoregions in which biodiversity and ecological function are at highest risk because of extensive habitat conversion and limited habitat protection (*11*); (ii) BHs are areas featuring exceptional concentrations of endemic species and experiencing exceptional loss of habitat (*12*); (iii) EBAs are areas that encompass the overlapping breeding ranges of restricted-range species, such that the complete ranges of two or more restricted-range species are entirely included within the boundary of the EBA (*13*); (iv) KBAs are globally important sites that are large enough or sufficiently interconnected to support viable populations of the species for which they are important (*14*); (v) CPDs are sites of global botanical importance based on their high plant endemism and species richness (*15*); (vi) G200s are large-scale priority areas of uniform ecological features, chosen for the conservation of the most outstanding and representative of the world’s habitats (*16*); and (vii) IFLs are unbroken expanses of natural ecosystems within the current forest extent, with no remotely detected signs of human activity, and large enough that all native biodiversity, including viable populations of wide-ranging species, could be maintained. IFLs have high conservation value and are critical for stabilizing terrestrial carbon storage, harboring biodiversity, regulating hydrological regimes, and providing other ecosystem functions (*17*).

Because of the differences in the selection of surrogates, emphasis on the criteria, and designation methods, these templates are significantly different from each other (table S2). For example, as surrogates for biodiversity, CE and G200 focus on the ecoregion, EBA focuses on birds, BH and CPD focus on plants, and IFL focuses on forest landscapes, while KBA focuses on species and ecosystems. Vulnerability and irreplaceability are widely accepted as a fundamental criterion in the identification of conservation priorities (*51*–*53*). Irreplaceability reflects how important a specific area is for effective conservation, and vulnerability is about the sensitivity of particular biodiversity features (*52*). In these templates, EBA, CPD, G200, and IFL take irreplaceability into special consideration; CE stresses vulnerability, while BH and KBA stress both irreplaceability and vulnerability. As for the designation method, CE, BH, CPD, G200, and IFL are the products of top-down scientific research, while KBA and EBA are designated from the bottom-up. It is obvious that each template alone is not sufficient for biodiversity conservation and, therefore, an overlay analysis is required.

Spatial data for these templates are available online as vector (e.g., polygon) or raster format. To ensure the accuracy of area calculation, all data were projected to Eckert IV (*54*) and transformed into raster format at 1-km resolution.

### Identification of CEZs

To exclude unsuitable areas for PA designation and reduce conservation cost (*55*), we applied the data of LIA (*21*) in the identification of CEZs. Areas with lower human influence—wild or wilderness—contribute to important ecosystem service and biodiversity (*56*) and have typically been viewed as more feasible for PA designation. Among the latest studies on global human impact assessment including Human Footprint (*57*), Human Modification (*22*), and LIAs (*21*), we opted to use LIA for two main reasons. First, compared with other assessments, LIA uses more recent data. Second, LIA uses the Boolean overlay method and, so, creates polygons with clearly defined boundaries. Together, these provide a more reliable platform for planning PA designation, while the segmentation of continuous Human Footprint and Human Modification would cause considerable uncertainty if applied at a global scale (*58*). We identified CEZs as lands that lie in both CPZs and LIAs. CEZs are then categorized into three levels according to the levels of CPZ.

### Setting global and national PA coverage targets

To propose national PA targets, a gap analysis was conducted by identifying areas currently within CEZs but not covered by existing PAs. PA targets are defined at three levels: (i) ambitious target, requiring all unprotected CEZs to be added into PA systems; (ii) moderate target, requiring unprotected level 1 and level 2 CEZs to be added into PA systems; and (iii) conservative target, requiring only unprotected level 1 CEZs to be covered by PAs. To assist with the planning of conservation actions, unprotected level 1 CEZs should be prioritized for protection, followed by unprotected level 2 and level 3 CEZs. The three targets were calculated by Eqs. 1 to 3

$$T_{C}=\frac{\text{PA}+{\text{CEZ}}_{u1}}{A}$$ (1)

$$T_{M}=\frac{\text{PA}+{\text{CEZ}}_{u1}+{\text{CEZ}}_{u2}}{A}$$ (2)

$$T_{A}=\frac{\text{PA}+{\text{CEZ}}_{u1}+{\text{CEZ}}_{u2}+{\text{CEZ}}_{u3}}{A}$$ (3)

where *T*_C_ is the conservative target for the statistical unit, *T*_M_ is the moderate target, *T*_A_ is the ambitious target, CEZ_u1_ is the total area of unprotected level 1 CEZs, CEZ_u2_ is the total area of unprotected level 2 CEZs, CEZ_u3_ is the total area of unprotected level 3 CEZs, and *A* is the total area of that statistical unit. The statistical unit is global and includes each CBD party.

For current PAs, we used December 2019 data from WDPA that include 225,198 PAs (*38*). We only used terrestrial area data and adopted a conservative approach on selecting PAs to be included in our analysis. PAs less than 1 km^2^ were excluded. UNESCO Man and Biosphere Reserves and “undesignated” PAs were also excluded as their core conservation areas often overlap with other PAs. Point data were transformed into polygons using simple buffer zones according to area. In total, existing PAs cover 14.1% of the global terrestrial area (excluding Antarctica and Greenland).

## SUPPLEMENTARY MATERIALS

Supplementary material for this article is available at http://advances.sciencemag.org/cgi/content/full/6/37/eabc3436/DC1
